# Immunoexpression of TS, p53, COX2, EGFR, MSH6 and MLH1 biomarkers and its correlation with degree of differentiation, tumor staging and prognostic factors in colorectal adenocarcinoma: a retrospective longitudinal study

**DOI:** 10.1590/1516-3180.2018.0270071218

**Published:** 2019-05-08

**Authors:** Wilson Roberto Batista, Gianni Santos, Flávia Maria Ribeiro Vital, Delcio Matos

**Affiliations:** I MD. Coordinator, Oncological Surgery Sector, Muriaé Cancer Hospital, Muriaé (MG), Brazil, and Doctoral Student, Universidade Federal de São Paulo (UNIFESP), São Paulo (SP), Brazil.; II MSc. Statistician, Applied Statistics Sector, Pro-Rectorate for Postgraduate Studies and Research, Universidade Federal de São Paulo (UNIFESP), São Paulo (SP), Brazil.; III MD. Physiotherapist, Physiotherapy Sector, Muriaé Cancer Hospital, Muriaé (MG), Brazil.; IV MD, MSc, PhD. Associate Professor, Department of Surgery, Escola Paulista de Medicina (EPM), Universidade Federal de São Paulo (UNIFESP), São Paulo (SP), Brazil.

**Keywords:** Colorectal neoplasms, Biomarkers, Neoplasms, Forecasting

## Abstract

**BACKGROUND:**

There are cases of colorectal tumors that, although small, show more aggressive evolution than large tumors. This motivated us to study whether there are any proteins capable of alerting about these changes. The aim here was to correlate the immunoexpression of the TS, p53, COX2, EGFR, MSH6 and MLH1 biomarkers in tumors in patients with colorectal adenocarcinoma, with the degree of cell differentiation, tumor staging and clinical-pathological prognostic factors.

**DESIGN AND SETTING:**

Retrospective observational study at a public tertiary-level hospital.

**METHODS:**

We analyzed tissue-microarray paraffin blocks of tumor tissues that had been resected from 107 patients. We used Fisher’s exact test to study associations between tumor differentiation/staging and the immunoexpression of biomarkers. We also used Kaplan-Meier estimation, the log-rank test and the adjusted Cox regression model to investigate the patients’ overall survival (in months) according to biomarkers and disease-free interval.

**RESULTS:**

The degree of tumor differentiation and tumor staging were not associated with the biomarkers, except in cases of patients in stages III or IV, in which there was a correlation with MLH1 expression (P=0.021). Patient survival and disease-free interval were not associated with the biomarkers.

**CONCLUSION:**

There were no associations between the degree of tumor differentiation, staging, length of survival or disease-free interval and the immunoexpression of the TS, *p53*, COX2, EGFR or *MSH6* tumor markers. In advanced cases of colorectal adenocarcinoma (stages III and IV), there was a higher percentage of *MLH1*-negative results.

## INTRODUCTION

Colorectal cancer (CRC) is considered to be the third most commonly diagnosed and the second greatest cause of mortality due to cancer in North America.^1^ In Brazil, similar data have been reported from research carried out by the National Cancer Institute (Instituto Nacional do Câncer, INCA). In 2018, the estimate incidence of CRC was 36,360 new cases (17,380 men and 18,980 women), with the highest incidence in the age group between 50 and 70 years.^2^ Approximately one in three people who develop the disease die of it.^3^

The most significant independent prognostic factor for CRC is the tumor-node-metastasis (TNM) stage and the “potential” residual disease after surgery.^4^ Neoplastic recurrence is a frequent cause of death among patients undergoing primary disease resection with curative intent,^5^^,^^6^ and this is one of the reasons for conducting further studies on CRC prognosis.

The *p53* tumor suppressor gene acts as a damage sensor in relation to deoxyribonucleic acid (DNA) and assists in the repair system using checkpoints to halt the cell cycle or induce apoptosis, thus preventing cell proliferation.^7^ In *p53*-mutation cells there is no DNA repair in the cell cycle.^8^ These genetically unstable cells tend to accumulate mutations, thereby leading to rapid proliferation of cell clones with mutated DNA, and thus to neoplastic transformation.^9^

Some authors agree that overexpression of epidermal growth factor receptors (EGFRs) is­associated with lower survival rates and worse prognosis.^10^ EGFRs are tyrosine kinase receptorsthat are involved in cascade-like activation, which leads to cell differentiation andmultiplication.^11^

The cyclo-oxygenase-2 (COX-2) enzyme plays a key role in conversion of arachidonic acid (AA) into prostaglandins, which have been associated with colorectal carcinogenesis.^12^ No conclusions have yet been reached regarding the relationship between COX-2 expression and patient survival.^13^

The major repair genes, i.e. MutS-homolog1 *(MLH1*), MutS-homolog6 *(MSH6),* MutS-homolog2 *(MSH2),* postmeiotic segregation increased 1 *(PMS1*) and postmeiotic segregation increased 2 (*PMS2*), play important roles in correcting mutations associated with oxidative stress. It is important to correct for addition of CH_3_ radicals to DNA bases.^14^^,^^15^^,^^16^ It has been shown that oxidative stress caused by oxygen free radicals breaks single and double DNA strands, thus inducing errors in the nitrogenous base pairs, which may lead to genetic mutations.^17^^,^^18^ Cells have defense mechanisms against these errors, consisting of DNA repair systems (i.e. mismatch repair, MMR).^16^ Deficiencies in this DNA repair mechanism constitute an important molecular pathway for CRC development, which occurs in the cases of approximately 15% of colorectal neoplasms.^19^

## OBJECTIVE

To study the immunoexpression of the TS, *p53*, COX2, EGFR*, MSH6* and *MLH1* biomarkers in patients with colorectal carcinoma; and to make correlations with the degree of cell differentiation, tumor staging and clinical-pathological prognostic factors.

## METHODS

### Study design, setting and ethics

We used tissues from patients who were operated at the Muriaé Cancer Hospital (Hospital do Câncer de Muriaé, HCM), in Muriaé, Minas Gerais, Brazil. We submitted the study protocol to the Ethics Committee of HCM and to the “Platform Brazil” Research Ethics Council (approval protocol number 347.449).

### Samples, participants and surgical procedures

We analyzed the tissue samples of a convenience sample of all consecutive patients with colonic or rectal cancer who underwent operations between January 2003 and November 2008 and whose paraffin blocks were stored at the archives of the HCM Department of Pathology.

The inclusion criteria for these patients were that they underwent surgical resection of the colon or rectum due to adenocarcinoma, without presence of any other severe chronic degenerative diseases that would impair survival assessment, and with subsequent follow-up at the outpatient clinic. The following patients were excluded: those with past neoplasms, those who underwent palliative surgery and those who died in the immediate postoperative period. Our final sample comprised 107 specimens. Weobtained data from the patients’ medical records and we tried to reach patients through phone calls, calling them for follow-up visits, so that there would not be any lack of information in the records.

Patients who underwent elective surgery had been staged preoperatively, and all of these patients were operated by a single team, with postoperative follow-up performed in the outpatient ward. Colorectal carcinoma was diagnosed by means of anorectal examination and tests such as flexible sigmoidoscopy, colonoscopy and abdominal computed tomography (CT) scan.

Among cases of emergency surgery performed because of acute intestinal obstruction, the diagnosis was made by means of clinical examination, abdominal x-ray and CT scan, and laparotomy. Among the elective patients, the diagnosis and preoperative staging were done using the following: a) clinical examination; b) ancillary tests, i.e. measurement of carcinoembryonic antigen (CEA), colonoscopy, chest radiography, abdominal and pelvic CT scans and abdominal ultrasonography; c) colorectal tumor biopsy; d) biopsy of the metastatic lesion, when suspected; and e) pathology examination on the surgical specimen.

Colonic or colorectal resection surgery was performed after checking for neoplastic spread into other organs and for any structures affected by the tumor. The surgery potentially included regional lymphadenectomy, respecting the tumor resection criteria. The patients were postoperatively followed up early on, with periodic clinical evaluations and with laboratory (CEA), radiological and endoscopic procedures, to check for any early disease recurrence.

### Tissue microarray analysis

We used tissue microarrays (TMAs) to study the immunoexpression of TS protein, *p53*, COX2, EGFR, *MSH6* and *MLH1*. Thecolorectal cancer tissues obtained from biopsies or surgical specimens were fixed in 10% formalin and were processed using the paraffin embedding method for histological analysis. Histological sections of thickness 3 mm were obtained from each block. The sections were stained with hematoxylin-eosin and were reviewed by two pathologists to confirm the diagnosis and reassess the histopathological findings. These evaluations helped select the parts of the specimens from which the cylinders of tissue used in the TMAs were taken. The TMA slides were subjected to five immunohistochemical reactions, following the specifications of the primary antibodies used for eachbiomarker.

The TMA methodology was as follows:

1. The area selected was marked out in the respective paraffinblock;
2. A drained space (“*casela*”) was created in the recipient block;
3. A tissue cylinder of 1 mm in diameter was extracted from the donor block of the respective area of interest that had previously been selected;
4. The tissue cylinder thus obtained was transferred from the donor block to the “*casela*” that had previously been created in the recipient block;
5. New positions within the recipient block were progressively reached (through movement measured in fractions of millimeters), in order to create a collection of tissue samples, following a matrix arrangement;
6. Final block quality was assessed for storage purposes.

### Variables studied

We investigated the immunoexpression of the following variables, and described patients according to them: TS protein, *p53*, COX2, EGFR, *MSH6* and *MLH1*; along with the degree of tumor differentiation, tumor staging, overall survival and disease-free interval.

Surgery was registered as curative (radical) or palliative, according to whether residual macroscopic neoplastic lesions were found to exist postoperatively in staging tests and according to the results from histopathological examination of the surgical specimen. Curative procedures require radical excision of the tumor with adequate surgical margins, considering its vascular pedicle and the largest number of adjacent lymph nodes, with no positive margins seen in pathological evaluation. Palliative surgery involves incomplete tumor resection, tumor bypassor just making a stoma without resecting the tumor.

Recurrence was defined as tumor recurrence in local structures or in remote organs as metastases that originated from the colorectal tumor. Recurrence was confirmed based on clinical examination, laboratory tests, radiological imaging and/or endoscopic views.

The disease-free interval was considered to be the period of postoperative time within which there was no detection of cancer recurrence in patients who underwent a supposedly curative procedure. Survival was defined as the time interval between surgery and death for certain patients, or between surgery and the last visit to the clinic or telephone contact. The staging of lesions was carried out in accordance with the tumor, node and metastasis (TNM) classification system.

### Statistical analysis

The statistical analysis was performed by an independent researcher. The data of interest were collected from the patients’ records. The statistical analysis on all the data collected in this study was done descriptively.

For the quantitative variables (numerical variables), we calculated some summary measurements, such as average, minimum, maximum and standard deviation values. The qualitative variables (categorized variables) were analyzed by calculating the absolute and relative frequencies (percentages).^20^ We performed inferential analyses to confirm or refute evidence that was found in the descriptive analysis, consisting of an extension of the Fisher exact test^21^ to study associations between the degree of tumor differentiation and the immunoexpression of TS, COX2, EGFR, *MLH1*, *MSH6* and *p53*; and between tumor staging and the immunoexpression of TS, COX2, EGFR, *MLH1*, *MSH6* and *p53*.

Kaplan-Meier estimates,^22^ the log-rank test^23^ and the adjusted Cox regression model were used to investigate the individuals’ overall survival (in months) according to their immunoexpression of TS, COX2, EGFR, MSH6, MLH1 and p53; and the disease-free interval of six subjects (in months) according to their immunoexpression of TS, COX2, EGFR, *MSH6*, *MLH1* and *p53*.

For inferential analysis purposes, we used the significance level α = 5%. We stored the data in Excel for Windows 2007 spreadsheets. For statistical analyses, we use the R statistical software, version 2.10.1.

## RESULTS

Our sample involved 107 individuals, comprising 50 females (46.7%) and 57 males (53.3%). The women’s average age was 64.3years, ranging from 43 to 90 years, with a standard deviation of 11.7 years. The male group had a mean age of 57.2 years, ranging from 24 to 86 years, with a standard deviation of 16.8 years.

The inferential results regarding the association between the degree of tumor differentiation and the immunoexpression of TS, COX2, EGFR, MLH1, MSH6 and p53 showed that the degree of tumor differentiation was not associated with the immunoexpression of TS (P = 0.138), COX2 (P = 0.428), EGFR (P = 0.103), *MSH6* (P = 0.876), *MLH1* (P = 0.792) or *p53* (P = 0.884).

### Subject clinical stage (CS) distribution according to TS expression

There was no statistically significant difference in the clinical staging of individuals, in relation to TS expression (P = 0.817). Similarly, there was no statistically significant difference in staging, in relation to the immunoexpression of COX2 (P = 0.842), EGFR (P = 0.344), MSH6 (P = 0.923), MLH1 (P = 0.021) or p53 (P = 0.666) (Table 1).

**Table 1.** Clinical stage distribution among the individuals with colorectal cancer according to immunoexpression of TS, COX2, EGFR, MLH1, MSH6 and p53

	Clinical stage						P
	0 (n = 2)	I (n = 16)	II (n = 27)	III (n = 46)	IV (n = 16)	Total (n = 107)	
TS							
Focal	2 (100.0%)	5 (31.3%)	5 (18.5%)	11 (23.9%)	7 (43.8%)	30 (28.0%)	0.817ª
Inconclusive	-	-	1 (3.7%)	3 (6.5%)	-	4 (3.7%)	
Intense	-	2 (12.5%)	2 (7.4%)	3 (6.5%)	1 (6.3%)	8 (7.5%)	
Moderate	-	2 (12.5%)	4 (14.8%)	7 (15.2%)	3 (18.8%)	16 (15.0%)	
Negative	-	7 (43.8%)	15 (55.6%)	22 (47.8%)	5 (31.3%)	49 (45.8%)	
COX2							
Focal	1 (50.0%)	6 (37.5%)	13 (48.1%)	16 (34.8%)	5 (31.3%)	41 (38.3%)	0.842a
Inconclusive	-	1 (6.3%)	1 (3.7%)	2 (4.3%)	-	4 (3.7%)	
Intense	-	2 (12.5%)	6 (22.2%)	6 (13.0%)	2 (12.5%)	16 (15.0%)	
Moderate	-	5 (31.3%)	6 (22.2%)	17 (37.0%)	7 (43.8%)	35 (32.7%)	
Negative	1 (50.0%)	2 (12.5%)	1 (3.7%)	5 (10.9%)	2 (12.5%)	11 (10.3%)	
EGFR							
0	1 (50.0%)	-	-	3 (6.5%)	1 (6.3%)	5 (4.7%)	0.344ª
1	-	2 (12.5%)	1 (3.7%)	1 (2.2%)	-	4 (3.7%)	
2	-	4 (25.0%)	4 (14.8%)	9 (19.6%)	6 (37.5%)	23 (21.5%)	
3	1 (50.0%)	9 (56.3%)	21 (77.8%)	31 (67.4%)	9 (56.3%)	71 (66.4%)	
Inconclusive	-	1 (6.3%)	1 (3.7%)	2 (4.3%)	-	4 (3.7%)	
*MSH6*							
Inconclusive	-	1 (6.3%)	1 (3.7%)	2 (4.3%)	-	4 (3.7%)	0.923ª
Negative	2 (100.0%)	12 (75.0%)	19 (70.4%)	37 (80.4%)	12 (75.0%)	82 (76.6%)	
Positive	-	3 (18.8%)	7 (25.9%)	7 (15.2%)	4 (25.0%)	21 (19.6%)	
*MLH1*							
Inconclusive	-	1 (6.3%)	-	2 (4.3%)	-	3 (2.8%)	0.021ª
Negative	-	-	1 (3.7%)	13 (28.3%)	4 (25.0%)	18 (16.8%)	
Positive	2 (100.0%)	15 (93.8%)	26 (96.3%)	31 (67.4)	12 (75.0%)	86 (80.4%)	
p53							
Inconclusive	-	-	1 (3.7%)	1 (2.2%)	-	2 (1.9%)	0.666ª
Negative	2 (100.0%)	10 (62.5%)	14 (51.9%)	33 (71.7%)	10 (62.5%)	69 (64.5%)	
Positive		6 (37.5%)	12 (44.4%)	12 (26.1%)	6 (37.5%)	36 (33.6%)	

### Clinical stage distribution of the subjects according to MLH1 expression

We found an association between stage and MLH1, such that the group of individuals in stages III or IV had a higher percentage of MLH1-negative results (28.3%) than did the subjects in stages 0, I or II (3.7%) (P = 0.021) (Table 1).

The inferential results from univariate analysis (log-rank test) revealed that overall survival was not associated with the immunoexpression of TS (P = 0.480), COX2 (P = 0.998), EGFR (P=0.600), MSH6 (P = 0.318), MLH1 (P = 0.798) or p53 (P = 0.695) (Table2). Even after disregarding the subjects with results that were classified as inconclusive, we were able to confirm that survival was not associated with the immunoexpression of TS (P=0.502), COX2 (P=0.989), EGFR (P = 0.424), MSH6 (P = 0.129), MLH1 (P=0.496) or p53 (P = 0.979).

**Table 2.** Summary of the overall survival time (months) among the individuals with colorectal cancer, according to immunoexpression of MLH1

MLH1	Summary measurements				
	n	Average	Median	Minimum	Maximum
Inconclusive					
No death	3	30.8	20.3	16.5	55.7
Death	1	1.6	1.6	1.6	1.6
Total	4	23.5	18.4	1.6	55.7
Negative					
No death	12	26.1	21.6	4.3	59.5
Death	6	11.1	7.4	2.2	23.9
Total	18	21.1	15.4	2.2	59.5
Positive					
No death	61	29.0	23.6	2.6	66.6
Death	23	11.5	9.5	0.9	29.5
Total	84	24.2	20.0	0.9	66.6
Total					
No death	76	28.6	23.5	2.6	66.6
Death	30	11.1	7.4	0.9	29.5
Total	106	23.6	19.7	0.9	66.6

The inferential results from univariate analysis (log-rank test) revealed that the subjects’ disease-free interval did not correlate with their immunoexpression of TS (P = 0.356), COX2 (P = 0.885), EGFR (P = 0.786), MSH6 (P = 0.178), MLH1 (P = 0.691) or p53 (P = 0.441). Even after disregarding the individuals with inconclusive results, we were able to confirm that the disease-free interval was not associated with the immunoexpression of TS (P = 0.228), COX2 (P = 0.796), EGFR (P = 0.661), MSH6 (P = 0.071), MLH1 (P = 0.448) or p53 (P = 0.442).

It was noteworthy that there was a tendency for MSH6-positive individuals to have longer disease-free intervals than those of their MSH6-negative counterparts.

We used the statistical methodology of the multiple Cox regression model to confirm the findings obtained from univariate analysis (log-rank test). The multivariate analysis confirmed the evidence obtained in the univariate analyses, in which individuals’ length of survival was not associated with their immunoexpression of TS (P=0.794), COX2 (P = 0.885), EGFR (P = 0.882), MSH6 (P=0.142), MLH1 (P = 0.788) or p53 (P = 0.556). Moreover, the subjects’ disease-free interval was not associated with their immunoexpression of TS (P = 0.481), COX2 (P = 0.756), EGFR (P = 0.843), MSH6 (P=0.085), MLH1 (P = 0.464) or p53 (P = 0.164).

It is important to highlight that, to achieve Cox regression analysis of greater stability, we disregarded the individuals with results that were deemed inconclusive.

## DISCUSSION

To explain our findings, one theory might be that the specimens contained in the paraffin blocks did not represent the invasive tumor. It is becoming increasingly common in the literature to see reports of different expressions of tumor markers in the same surgical specimen. It is important to remember that advanced tumors are almost always heterogeneous in nature (i.e. with randomly distributed tissues), and of variable sizes (such that large masses tend to more intensely express antigenic reactions). Theseproperties can lead to cells of different degrees of differentiation within the same lesion, which can greatly influence immunoreactivity. Considering this theory, one solution might be to perform immunohistochemical examinations on tumor tissue by means of microarrays, as performed in this study.

Another possible way to explain our findings is to suggest that there might be an association with the type of antibody used. Monoclonal antibody sensitivities may differ, depending on the type used: some might react better with the basal membranes of epithelial tissues, and others with the cell cytoplasm.

A further way to explain our findings concerning the immunohistochemical tests relates to the issue of statistical power, i.e. whether the sample has the power to demonstrate a significant difference when this exists. It is possible that larger samples, with consequently higher statistical power, could overcome this potential shortcoming. This, however, is only a hypothesis.

Conflicting results regarding the TS, p53, COX2, EGFR, MSH6 and MLH1 immune markers in CRC have been reported, and these can be explained by the different numbers of samples, the techniques used and the wide variation in methodologies used in the various studies. Patient selection, tissue processing, immunohistochemical techniques, result interpretations and statistical analyses have been quite variable.^24^ For these reasons, we were led to study the association between the immunoreactivity of the tumor markers TS, p53, COX2, EGFR, MSH6 and MLH1 in cases of colorectal cancer and the main clinical and pathological prognostic factors. These factors comprise recurrence, disease-free interval, survival, cell differentiation and staging. However, separate analysis on these markers showed that there were no associations with these prognostic factors, except in the group of patients at stages III or IV, in which there was a higher percentage of MLH1-negative individuals (28.3%) than in the group of individuals at stages 0, I or II (2.3%).

Recent studies have demonstrated that silencing of the MLH1 gene is related to development of errors associated with replication of CRC cells, as was depicted in a study on microsatellite instability.^25^ It was also found that MLH1 repair gene expression is higher in normal tissue than in cancer tissue, which demonstrated the importance of this gene in maintaining DNA integrity.^24^^,^^26^

## CONCLUSION

TS, COX2, EGFR, *MSH6*, *MLH1* and *p53* expression, as measured through immunohistochemical analysis, was not associated with the clinical-pathological factors of the patients with colorectal adenocarcinoma studied, except for *MLH1* in some cases. This marker showed a significant difference in expression between patients at stages III and IV and those at stages I to IV of colorectal adenocarcinoma.
